# Antibody Responses to Influenza Vaccination are Diminished in Patients With Inflammatory Bowel Disease on Infliximab or Tofacitinib

**DOI:** 10.1093/ecco-jcc/jjad182

**Published:** 2023-11-06

**Authors:** Zhigang Liu, James L Alexander, Kai Yee Eng, Hajir Ibraheim, Sulak Anandabaskaran, Aamir Saifuddin, Laura Constable, Rocio Castro Seoane, Claire Bewshea, Rachel Nice, Andrea D’Mello, Gareth R Jones, Sharmili Balarajah, Francesca Fiorentino, Shaji Sebastian, Peter M Irving, Lucy C Hicks, Horace R T Williams, Alexandra J Kent, Rachel Linger, Miles Parkes, Klaartje Kok, Kamal V Patel, Julian P Teare, Daniel M Altmann, Rosemary J Boyton, Ailsa L Hart, Charlie W Lees, James R Goodhand, Nicholas A Kennedy, Katrina M Pollock, Tariq Ahmad, Nick Powell

**Affiliations:** Department of Metabolism, Digestion and Reproduction, Imperial College London, London, UK; Department of Metabolism, Digestion and Reproduction, Imperial College London, London, UK; Department of Gastroenterology, Imperial College Healthcare NHS Trust, London, UK; Department of Gastroenterology, St Marks Hospital and Academic Institute, Gastroenterology, London, UK; Department of Metabolism, Digestion and Reproduction, Imperial College London, London, UK; Department of Metabolism, Digestion and Reproduction, Imperial College London, London, UK; Department of Gastroenterology, Imperial College Healthcare NHS Trust, London, UK; Department of Metabolism, Digestion and Reproduction, Imperial College London, London, UK; Department of Gastroenterology, St Marks Hospital and Academic Institute, Gastroenterology, London, UK; Department of Metabolism, Digestion and Reproduction, Imperial College London, London, UK; Department of Gastroenterology, St Marks Hospital and Academic Institute, Gastroenterology, London, UK; Department of Metabolism, Digestion and Reproduction, Imperial College London, London, UK; Department of Metabolism, Digestion and Reproduction, Imperial College London, London, UK; Exeter Inflammatory Bowel Disease and Pharmacogenetics Research Group, University of Exeter, Exeter, UK; Exeter Inflammatory Bowel Disease and Pharmacogenetics Research Group, University of Exeter, Exeter, UK; Department of Clinical Chemistry, Exeter Clinical Laboratory International, Royal Devon University Healthcare NHS Foundation Trust, Exeter, UK; Division of Medicine & Integrated Care, Imperial College Healthcare NHS Trust, London, UK; Department of Gastroenterology, Western General Hospital, NHS Lothian, Edinburgh, UK; Centre for Inflammation Research, The Queen’s Medical Research Institute, The University of Edinburgh, Edinburgh, UK; Department of Metabolism, Digestion and Reproduction, Imperial College London, London, UK; Department of Gastroenterology, Imperial College Healthcare NHS Trust, London, UK; Department of Surgery and Cancer, Imperial College London, London, UK; Nightingale-Saunders Clinical Trials & Epidemiology Unit [King’s Clinical Trials Unit], King’s College London, London, UK; Department of Gastroenterology, Hull University Teaching Hospitals NHS Trust, Hull, UK; Hull York Medical School, University of Hull, Hull, UK; Department of Gastroenterology, Guy’s and St Thomas’ NHS Foundation Trust, London, UK; School of Immunology & Microbial Sciences, King’s College London, London, UK; Department of Metabolism, Digestion and Reproduction, Imperial College London, London, UK; Department of Gastroenterology, Imperial College Healthcare NHS Trust, London, UK; Department of Metabolism, Digestion and Reproduction, Imperial College London, London, UK; Department of Gastroenterology, Imperial College Healthcare NHS Trust, London, UK; Department of Gastroenterology, King’s College Hospital, London, UK; The NIHR Bioresource, University of Cambridge, Cambridge, UK; The NIHR Bioresource, University of Cambridge, Cambridge, UK; Department of Gastroenterology, Cambridge University Hospitals NHS Trust, Cambridge, UK; Department of Gastroenterology, Bart’s Health NHS Trust, London, UK; Department of Gastroenterology, St George’s Hospital NHS Trust, London, UK; Department of Metabolism, Digestion and Reproduction, Imperial College London, London, UK; Department of Gastroenterology, Imperial College Healthcare NHS Trust, London, UK; Department of Immunology and Inflammation, Imperial College London, London, UK; Department of Infectious Disease, Imperial College London, London, UK; Lung Division, Royal Brompton and Harefield Hospitals, Guy’s and St Thomas’ NHS Foundation Trust, London, UK; Department of Gastroenterology, St Marks Hospital and Academic Institute, Gastroenterology, London, UK; Department of Gastroenterology, Western General Hospital, NHS Lothian, Edinburgh, UK; Centre for Inflammation Research, The Queen’s Medical Research Institute, The University of Edinburgh, Edinburgh, UK; Exeter Inflammatory Bowel Disease and Pharmacogenetics Research Group, University of Exeter, Exeter, UK; Department of Gastroenterology, Royal Devon University Healthcare NHS Foundation Trust, Exeter, UK; Exeter Inflammatory Bowel Disease and Pharmacogenetics Research Group, University of Exeter, Exeter, UK; Department of Gastroenterology, Royal Devon University Healthcare NHS Foundation Trust, Exeter, UK; Department of Infectious Disease, Imperial College London, London, UK; NIHR Imperial Clinical Research Facility and NIHR Imperial Biomedical Research Centre, London, UK; Exeter Inflammatory Bowel Disease and Pharmacogenetics Research Group, University of Exeter, Exeter, UK; Department of Gastroenterology, Royal Devon University Healthcare NHS Foundation Trust, Exeter, UK; Department of Metabolism, Digestion and Reproduction, Imperial College London, London, UK; Department of Gastroenterology, Imperial College Healthcare NHS Trust, London, UK

**Keywords:** JAK-inhibitor, anti-TNF, humoral immunity, immunisation

## Abstract

**Background and Aims:**

We sought to determine whether six commonly used immunosuppressive regimens were associated with lower antibody responses after seasonal influenza vaccination in patients with inflammatory bowel disease [IBD].

**Methods:**

We conducted a prospective study including 213 IBD patients and 53 healthy controls: 165 who had received seasonal influenza vaccine and 101 who had not. IBD medications included infliximab, thiopurines, infliximab and thiopurine combination therapy, ustekinumab, vedolizumab, or tofacitinib. The primary outcome was antibody responses against influenza/A H3N2 and A/H1N1, compared to controls, adjusting for age, prior vaccination, and interval between vaccination and sampling.

**Results:**

Lower antibody responses against influenza A/H3N2 were observed in patients on infliximab (geometric mean ratio 0.35 [95% confidence interval 0.20–0.60], *p* = 0.0002), combination of infliximab and thiopurine therapy (0.46 [0.27–0.79], *p* = 0.0050), and tofacitinib (0.28 [0.14–0.57], *p* = 0.0005) compared to controls. Lower antibody responses against A/H1N1 were observed in patients on infliximab (0.29 [0.15–0.56], *p* = 0.0003), combination of infliximab and thiopurine therapy (0.34 [0.17–0.66], *p* = 0.0016), thiopurine monotherapy (0.46 [0.24–0.87], *p* = 0.017), and tofacitinib (0.23 [0.10–0.56], *p* = 0.0013). Ustekinumab and vedolizumab were not associated with reduced antibody responses against A/H3N2 or A/H1N1. Vaccination in the previous year was associated with higher antibody responses to A/H3N2. Vaccine-induced anti-SARS-CoV-2 antibody concentration weakly correlated with antibodies against H3N2 [*r* = 0.27; *p* = 0.0004] and H1N1 [*r* = 0.33; *p* < 0.0001].

**Conclusions:**

Vaccination in both the 2020–2021 and 2021–2022 seasons was associated with significantly higher antibody responses to influenza/A than no vaccination or vaccination in 2021–2022 alone. Infliximab and tofacitinib are associated with lower binding antibody responses to influenza/A, similar to COVID-19 vaccine-induced antibody responses.

## 1. Introduction

Patients with inflammatory bowel disease [IBD] are in a clinical risk group recommended for yearly seasonal influenza immunization. IBD, comprising ulcerative colitis [UC] and Crohn’s disease, are immune-mediated inflammatory disorders estimated to affect one in 125 people in the UK,^1^ with rising prevalence worldwide.^2^ Many patients with IBD require long-term immunosuppressive therapy to control inflammation, at the potential cost of increased susceptibility to infectious diseases, an issue which has been brought into sharp focus by the threat from the COVID-19 pandemic. The array of immunosuppressive treatments used in IBD includes immunomodulators [most commonly thiopurines], anti-cytokine therapies (including anti-tumour necrosis factor [anti-TNF] and anti-IL-12/23 drugs), anti-integrin therapies [vedolizumab], and small-molecule inhibitors of signalling (e.g. tofacitinib, a Janus Kinase [JAK] inhibitor). In addition to the risk of increased susceptibility to infection, immunosuppressive therapies may reduce the efficacy of immunization against vaccine-preventable infections. Indeed, anti-TNF and JAK inhibitors are associated with reduced vaccine-induced antibody-binding and viral neutralization following SARS-CoV-2 [severe acute respiratory syndrome coronavirus 2] vaccination and anti-TNF therapy is associated with increased risk of post-vaccination breakthrough infection.^3–6^

Influenza is an acute viral infection which attacks the ciliated epithelial cells in the upper or lower respiratory tract.^7^ In most cases the illness is self-limiting, but influenza remains a particular threat to vulnerable individuals, including people who are immunocompromised, with an estimated 650 000 global deaths per year.^8^ Patients with IBD are at higher risk of influenza and influenza-related complications including hospitalization.^9^ As of January 13, 2023, the Centers for Disease Control and Prevention estimated that there have been at least 24 million illnesses, 260 000 hospitalizations, and 16 000 deaths from influenza in the USA this season, with over 98% of laboratory-confirmed cases caused by influenza A viruses H3N2 and H1N1.^10^

Upon influenza virus infection, both the innate and adaptive immune systems are activated.^11^ The innate system, involving cells such as macrophages and neutrophils, quickly initiates a defensive response, recruiting additional immune cells via cytokines and chemokines. Subsequently, the adaptive immune response is engaged. B cells produce antibodies to neutralize the virus, while T cells assist B cells, and cytotoxic T cells eliminate infected cells. Vaccination against influenza aims to stimulate this adaptive response without causing severe infection. The vaccine introduces antigens, derived from a weakened, killed, or fragmented virus, into the body. This triggers an immune response, leading to the production of memory B and T cells that respond more efficiently to future infections. The immune response to vaccination is similar to natural infection but without the disease symptoms. However, response strength and duration vary depending on the vaccine, individual immune health, age, and other factors. As the influenza virus frequently mutates, the vaccine is updated annually, necessitating yearly vaccination for maintained protection. Annual influenza vaccination is recommended for patients with IBD receiving immunosuppressive therapies.^12^ Whilst immune responses to SARS-CoV-2 vaccination have been the subject of intensive research over the last 2 years, responses to influenza vaccination are less well characterized, particularly in patients on newer agents used in IBD. Previous studies have shown that the anti-TNF treatment infliximab is associated with reduced immunogenicity to influenza vaccination.^13–16^ However, data on responses to influenza vaccination in patients with IBD receiving thiopurine monotherapy, ustekinumab [anti-IL-12/23], vedolizumab, and tofacitinib are scarcer. Here, we aimed to determine relative antibody responses against influenza in patients receiving seasonal influenza vaccination whilst on the spectrum of key immunosuppressive drug regimens commonly used in IBD.

## 2. Materials and Methods

### 2.1. Study design and participants

This study included participants recruited to the VIP study [SARS-CoV-2 Vaccination immunogenicity in Immunosuppressed inflammatory bowel disease Patients], which is a UK multi-centre prospective observational study [registration number: ISRCTN13495664] aiming to evaluate the immunogenicity of COVID-19 vaccination in IBD patients on six different immunosuppressive treatment regimens [infliximab, thiopurine, infliximab and thiopurine combination therapy, ustekinumab, vedolizumab, or tofacitinib]. Participant recruitment, and inclusion and exclusion criteria have been described previously.^3^ In addition to the pre-specified inclusion criteria for our COVID-19 vaccine response analyses, additional inclusion criteria were applied for the current influenza vaccine response analysis as follows. Participants were surveyed prospectively on whether they received vaccination against influenza in the 2020–2021 and/or the 2021–2022 influenza seasons and the date of vaccination was recorded. Only participants who responded positively or negatively to whether they received vaccination against influenza in the 2021–2022 season were included in the analysis. Participants who did not respond to this question were excluded. In the 2021–2022 season, influenza vaccination in the UK was with a quadrivalent vaccine containing an A/Victoria/2570/2019 [H1N1] pdm09-like virus or an A/Wisconsin/588/2019 [H1N1] pdm09-like virus; an A/Cambodia/e0826360/2020 [H3N2]-like virus; a B/Washington/02/2019 [B/Victoria lineage]-like virus; and a B/Phuket/3073/2013 [B/Yamagata lineage]-like virus. Demographics were recorded as variables: age, gender, ethnicity, comorbidities, height and weight, smoking status, postcode, IBD disease activity (defined by patient-reported outcomes [PRO2]),^17,18^ and date of blood collection. Data were entered electronically into a purpose-designed REDCap database hosted at the Royal Devon University Healthcare NHS Foundation Trust.^19^ Participants without access to the internet or electronic device completed their questionnaires on paper case record forms that were subsequently entered by local research teams. Blood was collected from participants at two time points: 53–92 days after the second COVID-19 vaccine dose and 28–49 days after the third COVID-19 vaccine dose. The study protocol is available online [https://www.vipstudy.uk]. In the influenza analysis, we included participants who had a serum sample taken between 7 and 90 days after influenza vaccination in the 2021–2022 season.

### 2.2. Antibody measurement

Antibody binding responses against four influenza strains [A/Hong Kong H3N2, A/Michigan H1N1, B/Phuket HA, and B/Brisbane HA] were measured with a multiplexed Meso Scale Discovery [MSD] immunoassay [cat. no: K15365U, MSD]. Multiplex MSD assays were performed as per the manufacturer’s instructions [detailed in the Supplementary Material]. Viral neutralizing responses were not measured in this study.

We previously measured the binding antibody concentrations against SARS-CoV-2 wild-type virus in the VIP cohort.^4^ Here we performed Spearman correlation analysis between antibodies against SARS-CoV-2 wild-type virus and each of the influenza strains measured in this study.

### 2.3. Outcome measures

Our primary outcome was antibody responses against A/H3N2 and A/H1N1 influenza viruses 7–90 days after vaccination, stratified by baseline immunosuppressive therapy compared to healthy controls, adjusting for age, vaccination against influenza in the previous [2020–2021] season, and the interval between vaccination and blood sampling. Secondary outcomes included antibody responses in influenza-vaccinated vs unvaccinated individuals, antibody responses against influenza B viruses, and correlations between responses to influenza vaccination and COVID-19 vaccination.

### 2.4. Statistics

A statistical analysis plan was approved by the Study Management Group [available at https://www.vipstudy.uk/info]. Analyses were undertaken using R 4.1.0 [R Foundation for Statistical Computing]. Values of *p* < 0.05 with two-tailed tests were considered significant. We included patients with missing clinical data in analyses for which they had data and specified the denominator for each variable. Antibody concentrations are reported as the geometric mean and standard deviation. Other continuous data are reported as a median and interquartile range, and discrete data as numbers and percentages, unless otherwise stated. For the univariate analysis comparing antibody levels among groups, a Kruskal–Wallis test was performed to test significance.

Multivariable linear regression models were used to identify factors independently associated with antibody concentrations. Backward stepwise regression was used to test whether these variables were covariates: age, gender, ethnicity, body mass index [BMI], height, weight, smoking, IBD subtype, IBD disease activity (defined by patient-reported outcomes [assessed by PRO2 score]),^17,18^ vaccination against influenza in the previous [2020–2021] season, and interval in days between vaccination and blood sampling. Results are presented after exponentiation so that the model’s coefficients correspond to the geometric mean ratio associated with each covariate. The linearity, homogeneity of variance, collinearity, influential observations, and normality of residuals of each multivariate model were tested in R. Spearman correlation analysis was performed between antibodies against SARS-CoV-2 wild-type virus and each of the influenza strains measured in this study. We also performed a Spearman correlation analysis between the antibody concentration with days after vaccination to show the antibody decay against time.

### 2.5. Ethical considerations and role of funders

VIP is an investigator-led UK National Institute for Health Research COVID-19 study. Financial support was provided as an independent research grant by Pfizer Ltd. Pfizer Ltd had no role in study design, data collection or analysis, writing, or decision to submit for publication. Participants were included after providing informed, written consent. The Wales Research Ethics Committee 5 approved the study [REC reference 21/WA/0105] in March, 2021. The study was registered with the ISRCTN [No: 13495664] registry, and the protocol is available online at https://www.vipstudy.uk.

## 3. Results

Between May 28, 2021 and March 29, 2022, 561 adult individuals were recruited to the VIP study, of whom 266 [213 IBD, 53 healthy controls; 166 vaccinated against influenza in the 2021–2022 season and 100 unvaccinated] were eligible for inclusion in the current analysis. Patients with IBD were established for at least 12 weeks on immunosuppressive treatment regimens, including infliximab [*n* = 39], thiopurines [*n* = 42], infliximab and thiopurine combination therapy [*n* = 38], ustekinumab [*n* = 34], vedolizumab [*n* = 38], or tofacitinib [*n* = 22]. Participant characteristics are shown in Table 1. Self-reported rates of influenza vaccination were ≥60% across the six groups of patients with IBD.

**Table 1.** Demographics of the cohort in this study. Continuous variables are presented as median [IQR]. The other variables are presented as percentages within each group.

Characteristics	Control [*N* = 53, 20%]	Infliximab [*N* = 39, 15%]	Infliximab + thiopurine [*N* = 38, 14%]	Thiopurine [*N* = 42, 16%]	Tofacitinib [*N* = 22, 8%]	Ustekinumab [*N* = 34, 13%]	Vedolizumab [*N* = 38, 14%]	*p*-value
Age	40.80 [32.60, 52.10]	47.20 [35.30, 55.95]	39.10 [31.80, 53.80]	45.25 [37.73, 56.70]	46.00 [37.90, 54.72]	45.30 [34.58, 55.80]	45.50 [37.45, 61.57]	0.23
Gender								0.0060
Female	35 [66.04%]	15 [38.46%]	18 [47.37%]	25 [60.98%]	6 [27.27%]	18 [52.94%]	13 [34.21%]	
Male	18 [33.96%]	24 [61.54%]	20 [52.63%]	16 [39.02%]	16 [72.73%]	16 [47.06%]	25 [65.79%]	
Ethnicity								0.83
Non-white	8 [15.09%]	8 [20.51%]	8 [21.05%]	9 [21.95%]	4 [18.18%]	6 [17.65%]	11 [28.95%]	
White	45 [84.91%]	31 [79.49%]	30 [78.95%]	32 [78.05%]	18 [81.82%]	28 [82.35%]	27 [71.05%]	
BMI	23.57 [21.76, 25.76]	25.15 [23.21, 27.16]	24.63 [21.61, 26.66]	23.88 [22.09, 26.88]	25.53 [23.35, 28.04]	25.37 [22.72, 29.61]	24.76 [22.19, 29.46]	0.22
Diagnosis								0.00050^a^
CD	0	27 [69.23%]	21 [55.26%]	22 [52.38%]	0	33 [97.06%]	18 [47.37%]	
IBDU	0	2 [5.13%]	2 [5.26%]	1 [2.38%]	0	0	1 [2.63%]	
UC	0	10 [25.64%]	15 [39.47%]	19 [45.24%]	22 [100%]	1 [2.94%]	19 [50%]	
PRO2 active disease	0	1 [2.56%]	1 [2.63%]	3 [7.14%]	2 [9.09%]	3 [8.82%]	5 [13.16%]	0.067
Smoking								0.29
Currently	1 [1.89%]	2 [5.13%]	2 [5.26%]	1 [2.44%]	2 [9.09%]	2 [5.88%]	5 [13.16%]	
Not currently	14 [26.42%]	9 [23.08%]	12 [31.58%]	14 [34.15%]	11 [50%]	13 [38.24%]	10 [26.32%]	
Never	38 [71.70%]	28 [71.79%]	24 [63.16%]	26 [63.41%]	9 [40.91%]	19 [55.88%]	23 [60.53%]	
Days after vaccination	49.00 [42.00, 74.00]	42.00 [31.00, 54.00]	40.00 [29.00, 47.00]	44.00 [33.00, 66.00]	29.00 [21.50, 45.00]	34.50 [22.00, 46.25]	36.00 [29.00, 55.00]	0.0070
Vaccinated participants in 2020–2021	14 [26.41%]	22 [56.41%]	22 [57.89%]	23 [54.76%]	11 [50.00%]	17 [50.00%]	18 [47.37%]	0.029
Vaccinated participants in 2021–2022	31 [58.49%]	25 [64.10%]	25 [65.79%]	27 [64.29%]	11 [50.00%]	22 [64.71%]	25 [65.79%]	0.89
Heart disease	0	1 [2.63%]	0	1 [2.44%]	0	0	2 [5.26%]	0.41
Lung disease	3 [5.77%]	6 [15.79%]	4 [10.53%]	2 [4.88%]	3 [13.64%]	3 [9.09%]	3 [7.89%]	0.63
Kidney disease	0	2 [5.26%]	0	0	0	0	1 [2.63%]	0.17
Diabetes	0	2 [5.26%]	0	2 [4.88%]	0	2 [6.06%]	2 [5.26%]	0.28
Cancer	0	1 [2.63%]	0	1 [2.44%]	0	0	1 [2.63%]	0.75

Kruskal–Wallis rank sum test and Fisher’s exact test were used to test the significance. BMI, body mass index; CD, Crohn’s disease; UC, ulcerative colitis; IBDU, inflammatory bowel disease unclassified.

^a^Fisher’s test was performed among all groups excluding healthy controls.

**Figure 1.**
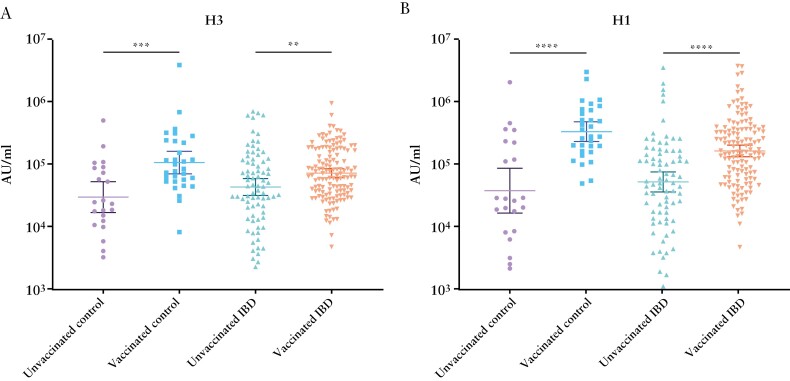
Antibody responses in healthy controls and patients with IBD against [A] A/H3N2 and [B] A/H1N1 [*n* = 266]. Geometric means are shown with 95% confidence intervals. **p < 0.01; ***p < 0.001; ****p < 0.0001.

Influenza vaccine-induced antibody levels in patients with IBD and healthy controls were measured by means of MSD analysis and those self-reporting influenza vaccination were compared with those without vaccination in the 2021–2022 season [Figure 1]. Against A/Hong Kong H3N2 vaccinated patients with IBD had higher geometric mean [GM] antibody concentrations (GM 71 370 AU/mL [95% CI 60 421, 84 302]) than unvaccinated patients with IBD (42 736 AU/mL [31 315, 58 323], *p* = 0.0068). Vaccinated healthy controls (105 289 AU/mL [69 521,159 459]) also had higher GM antibody concentrations against A/Hong Kong H3N2 than unvaccinated controls (29 481 AU/mL [16 692, 52 072], *p* = 0.0003). Against A/Michigan H1N1 vaccinated patients with IBD had higher GM antibody concentrations (165 312 AU/mL [133 482, 204 732]] than unvaccinated patients with IBD [53 108 AU/mL [36 755, 76 736], *p* < 0.0001). Vaccinated healthy controls (334 541 AU/mL [232 892, 480 556]) also had higher GM antibody concentrations against A/Michigan H1N1 than unvaccinated controls (38 534 AU/mL [16 942, 87 647], *p* < 0.0001). GM antibody concentrations against B Phuket and B Brisbane were also significantly higher in vaccinated patients with IBD than unvaccinated patients and in vaccinated controls than unvaccinated controls [Supplementary Figure S1; all *p* values <0.05].

**Figure 2.**
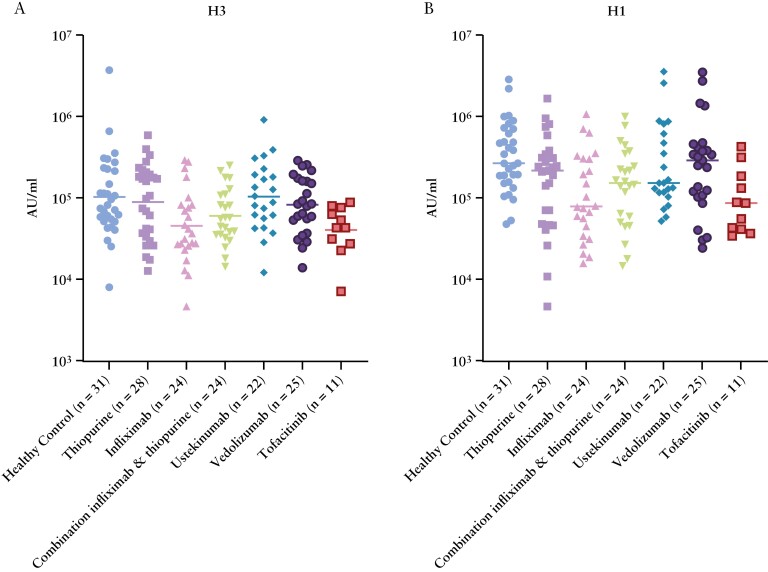
Unadjusted vaccine-induced antibody responses against [A] A/H3N2 and [B] A/H1N1 in patients with inflammatory bowel disease who received influenza vaccination in the 2021–2022 season, stratified by treatment group. Horizontal lines indicate geometric means.

We then investigated how different immunosuppressive treatment regimens in patients with IBD impacted unadjusted influenza vaccine-induced antibody responses. Including 166 participants who were sampled 7–90 days after receiving the 2021 flu vaccine, different treatments displayed distinct attenuation in antibody responses [Figure 2]. Patients treated with infliximab (GM 46 475 [95% CI 29 940, 72 142], *p* = 0.0056) or tofacitinib (41 198 [25 178, 67 411], *p* = 0.013) had significantly lower GM antibody concentrations against A/Hong Kong H3N2 relative to healthy controls (105 289 [69 521, 159 459]; Figure 2A). Patients treated with infliximab (109 202 [65 370, 182 423], *p* = 0.0008), thiopurine (164 733 [97 489, 278 359], *p* = 0.049), combination of infliximab and thiopurine (141 684 [87 849, 228 509], *p* = 0.012), and tofacitinib (94 322 [52 129, 170 665], *p* = 0.0016) had significantly lower GM antibody concentrations against A/Michigan H1N1 relative to controls (334 541 [232 892, 480 556]; Figure 2B). Unadjusted vaccine-induced antibody concentration data against B strains, stratified by IBD treatment regimen, are shown in Supplementary Figure S2.

**Figure 3.**
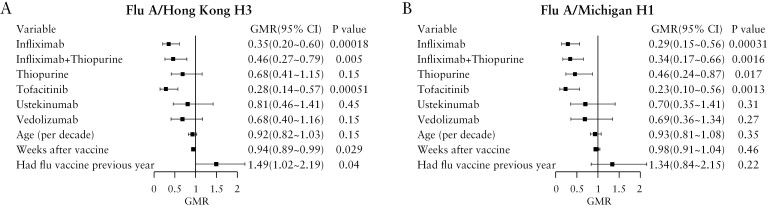
Multivariable models of vaccine-induced antibody responses against [A] A/H3N2 and [B] A/H1N1 in patients with inflammatory bowel disease who received influenza vaccination in the 2021–2022 season, stratified by treatment group [*n* = 166]. GMR, geometric mean ratio.

Time between influenza vaccination and blood sampling in this study was variable across the cohort. However, analysis of correlations between influenza vaccine-induced antibody concentrations and time in days between vaccination and blood sampling did not indicate a significant effect of antibody decay over the 83-day sampling window [Supplementary Figure S3]. Next, we performed multivariable linear regression to determine whether adjusting for participant age [a well-established factor in diminishing immune responses to vaccination], prior vaccination against influenza in the 2020–2021 season and time between vaccination and blood sampling impacted influenza vaccine-induced immune responses [Figure 3]. Lower antibody responses against A/Hong Kong H3N2 were observed in patients with IBD on infliximab (geometric mean ratio [GMR] 0.35 [95% CI 0.20–0.60], *p* = 0.0002), combination of infliximab and thiopurine therapy (0.46 [0.27–0.79], *p* = 0.0050), and tofacitinib (0.28 [0.14–0.57], *p* = 0.0005) [Figure 3A]. Thiopurine monotherapy (0.68 [0.41–1.15], *p* = 0.15), ustekinumab (0.81 [0.46–1.41], *p* = 0.45), and vedolizumab (0.68 [0.40–1.16], *p* = 0.15) were not associated with lower antibody responses against H3N2. A longer interval between vaccination and sampling was associated with lower antibody concentrations against A/Hong Kong H3N2 (0.94 [0.89, 0.99], *p* = 0.029), and prior vaccination against influenza in the previous 2020–2021 season was associated with higher antibody concentrations (1.49 [1.02–2.19], *p* = 0.040). Reduced antibody responses against A/Michigan H1N1 were observed in patients on infliximab (0.29 [0.15–0.56], *p* = 0.0003), combination of infliximab and thiopurine therapy (0.34 [0.17–0.66], *p* = 0.0016), thiopurine monotherapy (0.46 [0.24–0.87], *p* = 0.017), and tofacitinib (0.23 [0.10–0.56], *p* = 0.0013) relative to controls. Ustekinumab (0.70 [0.35–1.41], *p* = 0.31) and vedolizumab (0.69 [0.36–1.34], *p* = 0.27) were not associated with reduced antibody responses against A/Michigan H1N1 [Figure 3B]. We performed a sensitivity analysis to check if the presence of active disease had an impact on our models [Supplementary Figure S4]. Including active disease as a variable in the multivariable models did not alter the significant associations found between IBD therapies and antibody responses. Infliximab therapy was associated with reduced antibody responses against both B/Phuket and B/Brisbane and tofacitinib was associated with lower antibody responses against B/Phuket [Supplementary Figure S5].

**Figure 4.**
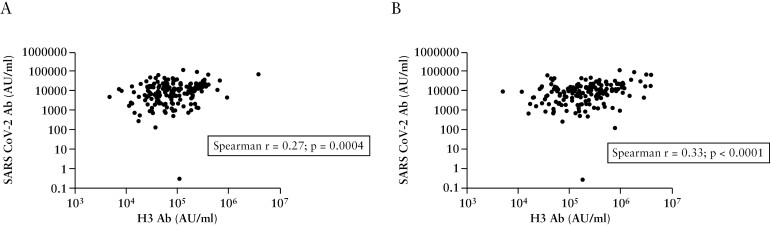
Spearman correlations of [A] A/H3N2 and [B] A/H1N1 vaccine-induced antibody responses [*x*-axis] vs SARS-CoV-2 vaccine-induced antibody responses [*y*-axis].

Previously, we reported antibody responses against SARS-CoV-2 wild-type virus in this cohort. Next, we performed a Spearman correlation analysis between SARS-CoV-2 binding antibody concentrations and influenza binding antibody concentrations in study participants who had received both influenza vaccination in the 2021–2022 season and the full primary schedule [three doses] of COVID-19 vaccination [Figure 4]. There was a statistically significant correlation between anti-SARS-CoV-2 antibody concentration and antibody concentrations of A/Hong Kong H3N2 [*r* = 0.27; *p* = 0.0004] and A/Michigan H1N1 [*r* = 0.33; *p* < 0.0001]. Similar correlations observed between anti-SARS-CoV-2 antibody concentrations and B/Phuket [*r* = 0.16; *p* = 0.034] and B/Brisbane [*r* = 0.17; *p* = 0.029] were more modest [Supplementary Figure S6]. Correlations between responses against influenza strains were overall stronger between different influenza strains than between corresponding responses against SARS-CoV-2 [Supplementary Figure S7].

## 4. Discussion

With the widespread use of immunosuppressive therapies for patients with IBD, it is important to understand how the off-target effects of these therapies might influence immune responses to common respiratory infections. We have previously shown that vaccine-induced humoral responses against SARS-CoV-2 are impaired in patients with IBD receiving infliximab or tofacitinib therapy, but are relatively preserved in patients receiving thiopurine monotherapy, ustekinumab, or vedolizumab.^3–5^

In the current study, we observed that influenza vaccine-induced antibody concentrations are diminished in patients receiving infliximab or combined infliximab and thiopurine therapy. TNF is a critical cytokine in multiple aspects of the immune response, including T-cell-dependent antibody production and the formation of B cell follicles in germinal centres.^20^ Thiopurines reduce intracellular synthesis of purines, leading to decreased circulating T and B cells and diminished immunoglobulin production.^21^ Our data corroborate other studies^13,15,16,22^ and suggest that the impact of anti-TNF therapy on vaccine-induced immunity may be manifested across antigen and vaccine platforms.^23^ In keeping with this concept, we found correlations between humoral responses to influenza and COVID-19 vaccinations, although the relative weakness of these correlations suggests that other factors are playing a role in modulating immune responses to different vaccine antigens, including genetics, nutrition,^24^ and the composition and function of the gut microbiota.^25,26^

We also observed diminished antibody responses against A/H1N1 and A/H3N2 in tofacitinib recipients. There are no existing studies on responses to influenza vaccination in tofacitinib-treated patients with IBD, although a trial in rheumatoid arthritis found that fewer patients treated with tofacitinib, as compared to placebo, developed protective vaccine-induced influenza antibody titres.^27^ Tofacitinib is a pan-JAK inhibitor with most activity against JAK1 and JAK3 signalling, which play a key role in innate and adaptive immune function, including B-cell responses.^28–30^ Ustekinumab [anti-IL12/23] and vedolizumab [anti-integrin] are biological therapies thought to have more favourable safety profiles and thus are increasingly used preferentially in older and co-morbid patients with IBD in whom the risk of infections is higher. Existing data relating to the impact of ustekinumab and vedolizumab on immune responses to influenza vaccination in IBD are scarce, limited to small studies including fewer than 20 patients on the respective treatment regimen of interest.^31–33^ There is limited mechanistic understanding on the effects of these two agents on immune responses to vaccination. Ustekinumab selectively inhibits IL-12 and IL-23, and might theoretically influence responses to influenza via its impact on the Th1 and Th17 pathways.^34^ Vedolizumab inhibits trafficking of T cells to the gut, and is not thought to impact on the immune response to systemically administered vaccines or infections outside the gut.^35^ Reassuringly, our data suggest that ustekinumab and vedolizumab do not have a significant impact on influenza vaccine-induced humoral responses.

The age-adjusted relative risk (Mantel–Haenszel age-adjusted rate ratio [RR], 95% CI) of death for influenza in immunosuppressed individuals is 47.3 [35.5–63.1].^36^ Vulnerable individuals, including patients with IBD taking immunosuppressive therapies, are advised to receive annual influenza vaccination to reduce their risk. Our data reinforce the existing guidance, showing that patients with IBD receiving vaccination against influenza in the 2021–2022 season mount significantly higher antibody responses to a range of influenza viral strains than do their unvaccinated counterparts. Moreover, vaccination in the previous 2020–2021 season was associated with higher responses to vaccination in the 2021–2022 season, suggesting that annual vaccination confers a significant boost in antibody responses over one-off vaccination. Yet rates of vaccination in eligible patients with IBD have historically been low at around a third, raising the question of how vaccination rates can be improved.^37^ Providing an IBD clinic on-site vaccination service has been shown to enhance vaccination rates,^38^ but there is also evidence that simple healthcare provider recommendation for vaccination is strongly associated with the likelihood of vaccine uptake.^37^

We acknowledge several limitations in our study. First, the VIP study was designed to investigate responses to COVID-19 vaccination. Thus, although our analysis adjusted for time interval to sampling and many participants received simultaneous influenza vaccination, blood samples were timed with respect to COVID-19 vaccine doses rather than influenza doses, leading to heterogeneity in sampling intervals across our cohort. Second, given the lack of baseline pre-vaccination samples, we cannot comment directly on the magnitude of humoral responses as a result of vaccination, although by comparing antibody concentrations between unvaccinated and vaccinated individuals [Figure 1], we are able to observe cross-sectional differences in antibody responses. Third, we relied on self-reported influenza vaccination status rather than healthcare record data to determine eligibility for this study, although studies have demonstrated that self-reported status is a reliable surrogate of gold-standard administrative record data.^39,40^ Fourth, we do not have data on influenza infections prior to or during the study period, nor influenza vaccinations before 2020, both of which might have influenced antibody concentrations. We note that rates of influenza infection were unusually low at the time of our study and thus unlikely to have been an important confounder. Fifth, the number of influenza-vaccinated patients was modest for each therapeutic sub-group, particularly for tofacitinib, limiting the confidence of our results. Finally, this study looked at humoral and not cell-mediated immunity, and our results are observational with no insights into the immunological mechanisms underlying our findings.

In conclusion, our study shows antibody responses to Influenza/A were higher in patients with IBD who received influenza vaccination in 2021–2022 than in those patients who did not receive vaccination, and vaccination in both the 2020–2021 and 2021–2022 seasons was associated with significantly higher responses than one-off vaccination in 2021–2022 alone. However, infliximab and tofacitinib are associated with lower binding antibody responses to Influenza/A, similar to COVID-19 vaccine-induced antibody responses, and we suggest adherence to guidelines advising annual vaccination against common respiratory infections is particularly important in patients with IBD receiving these treatments.

## Supplementary Material

jjad182_suppl_Supplementary_Material

## Data Availability

The data of this study are available under a transfer agreement from the corresponding author based on a reasonable request.
