# Supplementary material for: Antibody Responses to Influenza Vaccination are Diminished in Patients With Inflammatory Bowel Disease on Infliximab or Tofacitinib
Source: J Crohns Colitis. 2023 Nov 6;18(4):560–9. doi: 10.1093/ecco-jcc/jjad182 (PMC11037107; doi:10.1093/ecco-jcc/jjad182)
Supplement: jjad182_suppl_Supplementary_Material [file jjad182_suppl_supplementary_material.docx]

**Antibody responses to Influenza vaccination are diminished in inflammatory bowel disease patients on infliximab and tofacitinib**

**SUPPLEMENTARY DATA**

**SUPPLEMENTARY METHODS**

Antibody measurement using MSD Immunoassay

96-well plates were blocked with MSD Blocker for 1 hour. Plates were then washed in MSD washing buffer, and samples were diluted 1:50000 in the diluent buffer. Internal controls and standards were added to each plate. After 1 h incubation, recombinant human SULFO-TAG™ was added to all wells. After a further 1 h, plates were washed, and MSD GOLD™ Read Buffer B was added. Plates were then immediately read using a MESO® SECTOR S 600 Reader and antibody levels were calculated against the standard curve.

**SUPPLEMENTARY FIGURES**

**Figure S1** Antibody responses in healthy controls and patients with IBD against influenza B (n=266)

**Figure S2** Unadjusted vaccine-induced antibody responses in participants who received influenza vaccination in 2021-2022 season stratified by treatment group. Horizontal lines indicate the geometric means.

**Figure S3** Correlation of influenza vaccine antibody responses with days after vaccination (only participants sampled 7-90 days after 2021-2022 vaccine).


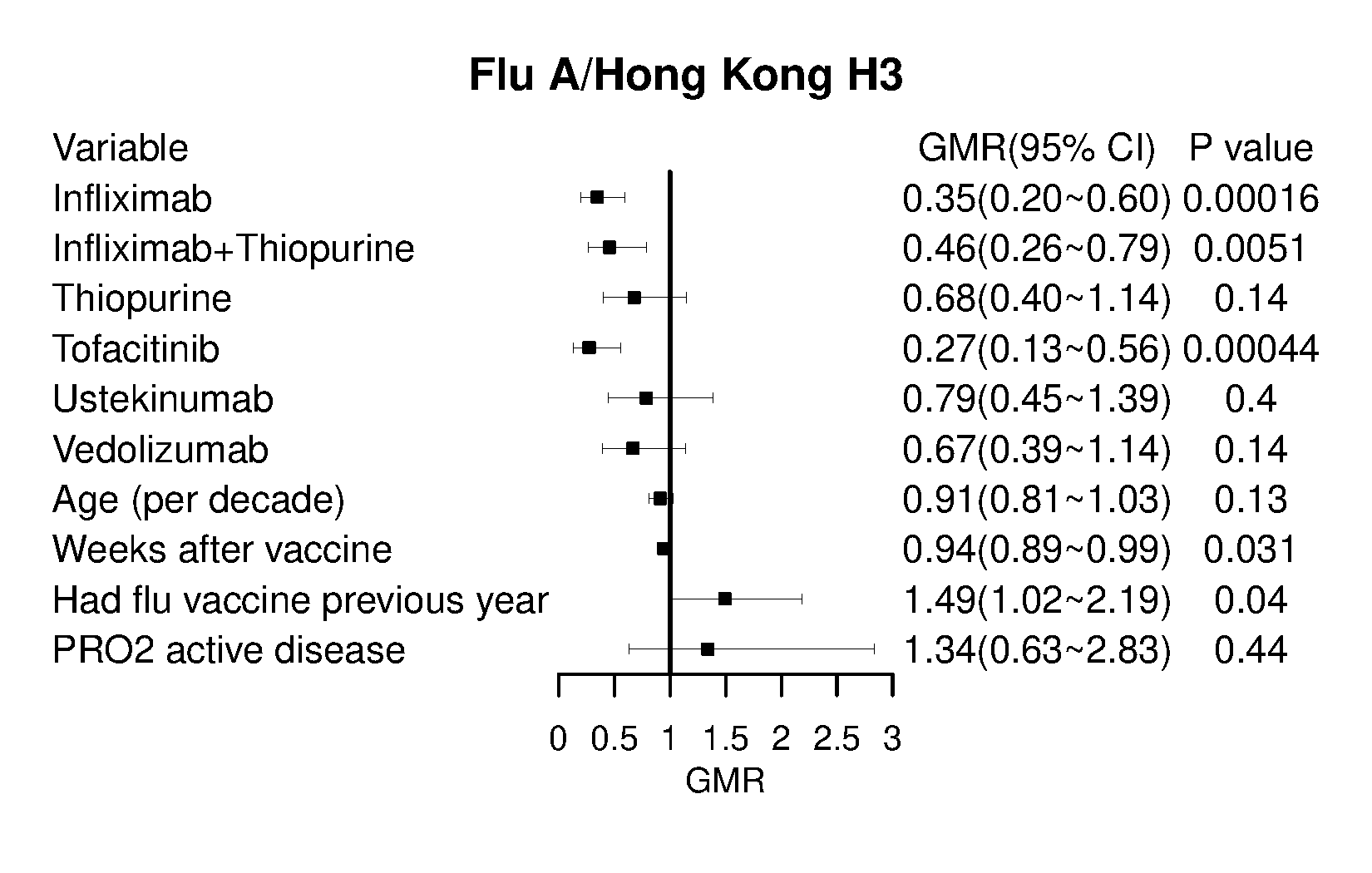

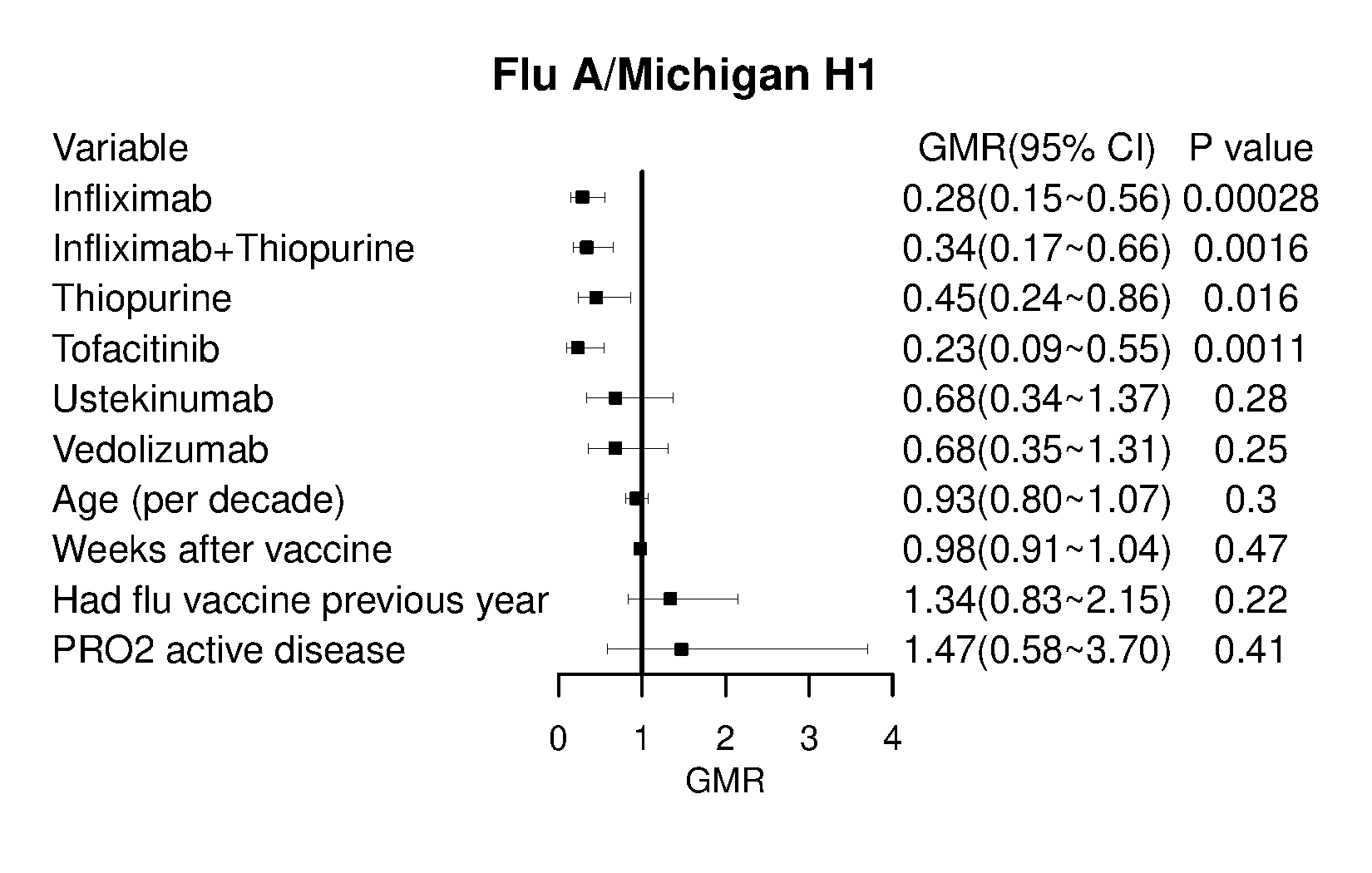


**Figure S4** Multivariable linear regression models of vaccine-induced antibody responses in participants who received influenza vaccination in 2021-2022 season stratified by treatment group, accounting for active disease (n=166).


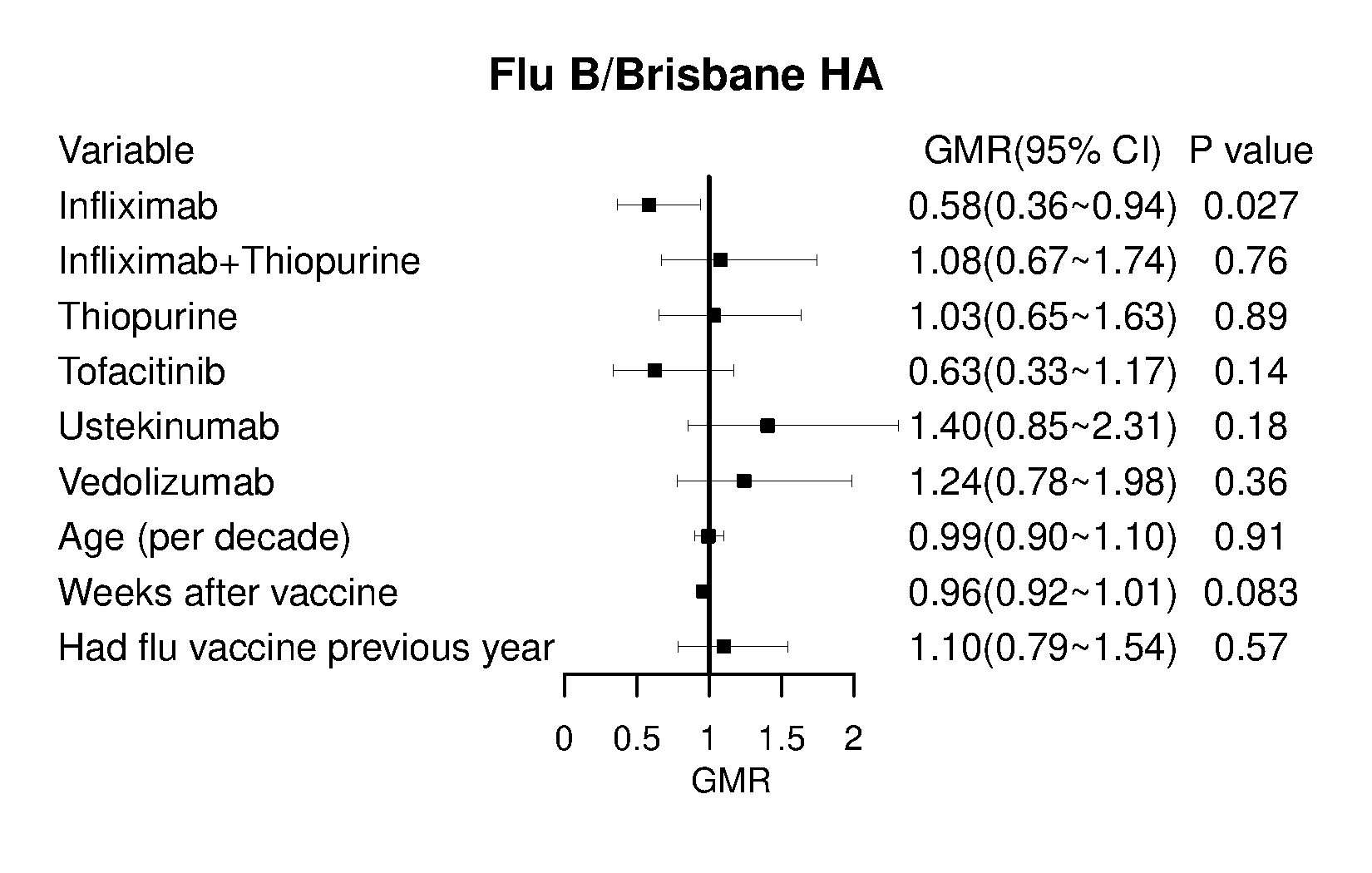

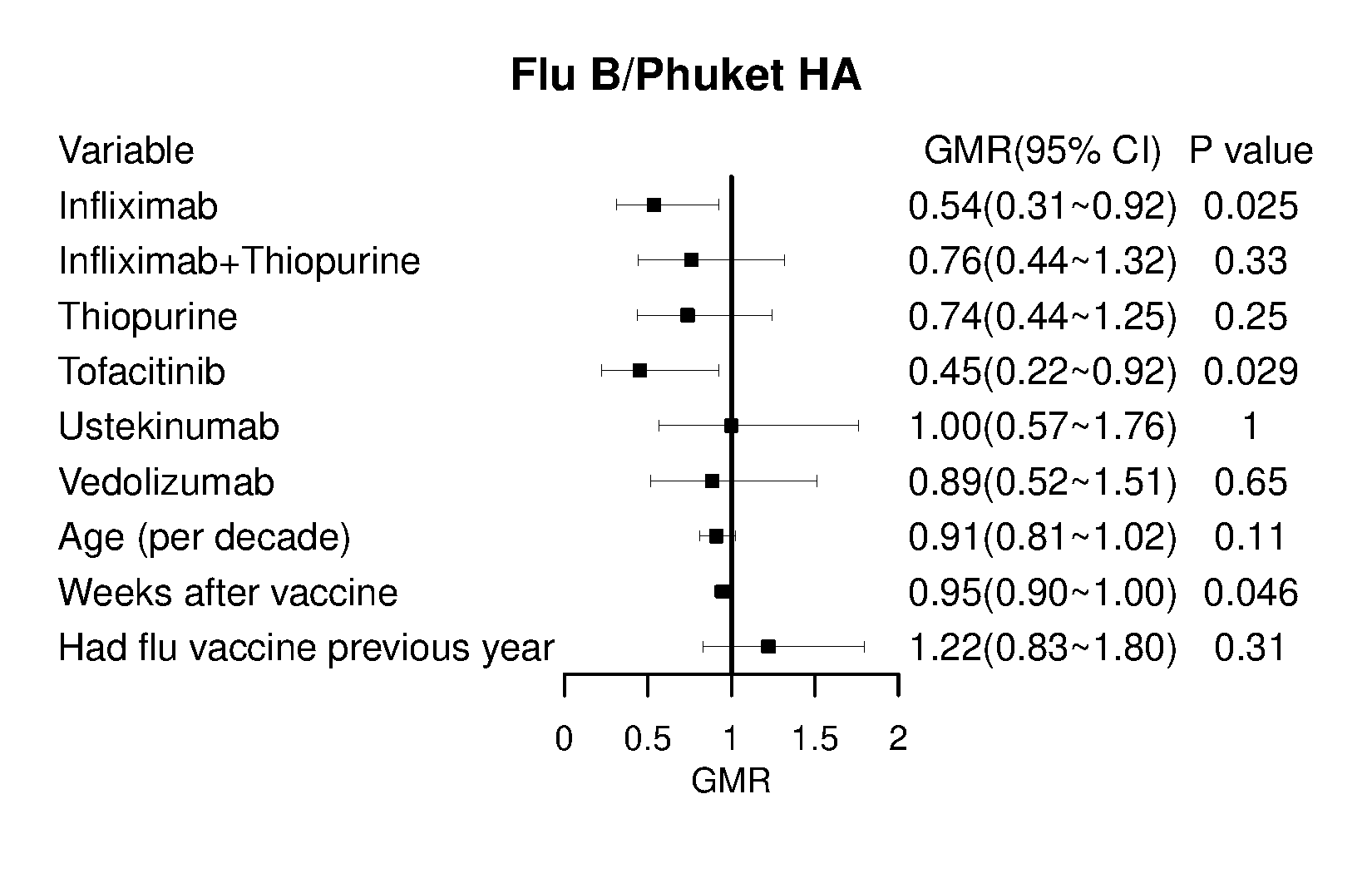


**Figure S5.** Multivariable linear regression models of vaccine-induced antibody responses in participants who received influenza vaccination in 2021-2022 season stratified by treatment group (n=116).

**Figure S6** Correlation of influenza vaccine antibody responses (x-axes) versus SARS-CoV-2 antibody responses (y-axes) (n=165)

**Figure S7** Correlation of influenza vaccine responses, strain versus strain (n=165)

**VIP INVESTIGATORS LIST**

| **First Name** | **Surname** |
| --- | --- |
| Ijeoma | Chukwurah |
| Sulaimaan | Haq |
| Parita | Shah |
| Stephanie | Wilken-Smith |
| Anitha | Ramanathan |
| Mikin | Patel |
| Lidia | Romanczuk |
| Rebecca | King |
| Jason | Domingo |
| Djamila | Shamtally |
| Vivien | Mendoza |
| Joanne | Sanchez |
| Hannah | Stark |
| Bridget | Knight |
| Louise | Bee |
| Charmaine | Estember |
| Anna | Barnes |
| Darcy | Watkins |
| Sam | Stone |
| John | Kirkwood |
| Marian | Parkinson |
| Helen | Gardner-Thorpe |
| Kate | Covil |
| Lauranne | Derikx |
| Beatriz | Gros Alcalde |
| Irish | Lee |
| Bessie | Cipriano |
| Giuseppe | Ruocco |
| Manisha | Baden |
| Graham | Cooke |
| Katrina | Pollock |
| Evgenia | Kourampa |
| Ciro | Pasquale |
| Elena | Robisco-Diaz |
| Suhaylah | Bhatti |
